# Interventions to prevent anastomotic leak after esophageal surgery: a systematic review and meta-analysis

**DOI:** 10.1186/s12893-020-01026-w

**Published:** 2021-01-18

**Authors:** Emma J. M. Grigor, Suha Kaaki, Dean A. Fergusson, Donna E. Maziak, Andrew J. E. Seely

**Affiliations:** 1 Department of Surgery, Division of Thoracic Surgery, The Ottawa Hospital, 501 Smyth Road, PO Box 201B, Ottawa, ON K1H 8L6 Canada; 2 Clinical Epidemiology Program, Ottawa Hospital Research Institute, Ottawa, Canada; 3 Faculty of Medicine, University of Ottawa, Ottawa, Canada

**Keywords:** Anastomotic leakage, Esophagectomy, Cancer, Carcinoma, Intervention, Adverse events

## Abstract

**Background:**

Anastomotic leakage (AL) is a common and serious complication following esophagectomy. We aimed to provide an up-to-date review and critical appraisal of the efficacy and safety of all previous interventions aiming to reduce AL risk.

**Methods:**

We searched MEDLINE and Embase from 1946 to January 2019 for randomized controlled trials (RCTs) evaluating interventions to minimize esophagogastric AL. Pooled risk ratios (RR) for AL were obtained using a random effects model.

**Results:**

Two reviewers screened 441 abstracts and identified 17 RCTs eligible for inclusion; 11 studies were meta-analyzed. Omentoplasty significantly reduced the risk of AL by 78% [RR: 0.22; 95% CI: 0.10, 0.50] compared to conventional anastomosis (3 studies, n = 611 patients). Early removal of NG tube significantly reduced the risk of AL by 62% [RR: 0.38; 95% CI: 0.02, 0.65] compared to prolonged NG tube removal (2 studies, n = 293 patients); Stapled anastomosis did not significantly reduce the risk of AL [RR: 0.92; 95% CI: 0.45, 1.87] compared to hand-sewn anastomosis (6 studies, n = 1454 patients). The quality of evidence was high for omentoplasty (vs. conventional anastomosis), moderate for early NG tube removal (vs. prolonged NG tube removal), and very low for stapled anastomosis (vs. hand-sewn anastomosis).

**Conclusions:**

This is the first meta-analysis to summarize the graded quality of evidence for all RCT interventions designed to reduce the risk of AL following esophagectomy. Our findings demonstrated that omentoplasty significantly reduced the risk of AL with a high quality of evidence. Although early NG tube removal significantly reduced AL risk, there is a need for further research to strengthen the quality of evidence for this finding. Evidence profiles presented in our review may help inform the development of future clinical practice recommendations.

*Systematic review registration:* CRD42019127181.

## Background

Esophagectomy is a critical component of curative treatment for esophageal cancer. This procedure carries a significant risk for certain adverse events among patients undergoing esophagectomy. One of the most serious adverse events associated with esophagectomy is anastomotic leakage (AL), which involves the leak of gastric fluid outside of the anastomosis postoperatively [1]. The presence of AL, with the rates being shown to occur up to 50% in some studies, is a potentially serious adverse event for patients and it has previously been significantly associated with prolonged length of stay, the formation of strictures, and increased morbidity and mortality [2, 3].

There have been several interventions conducted previously that aimed to prevent AL, ranging from surgical interventions to more conservative measures. Omentoplasty is a standardized surgical technique that harnesses a pedicle flap from the omentum (a layer of abdominal fat that is attached to the greater curvature of the stomach) to cover or wrap around the anastomosis site. The omental flap, secured in place with hand-sewn sutures, is well perfused by vascularity from the preserved left gastro-epiploic artery [4–6]. The improved vascularity and delivery of oxygenated and nutrient-rich blood to the surgical site from the omental flap is thought to enhance wound healing [7]. Omentoplasty has demonstrated promising findings in previous randomized controlled trial (RCT) studies to prevent AL after esophagectomy, which has been summarized in two previous meta-analyses conducted by Chen et al. 2014 and Yuan et al. 2019 [8, 9]. Another intervention investigated in previous studies includes the early removal of the nasogastric (NG) tube after esophagectomy. It is believed that increased strain on the wall of the esophagus during anastomotic site dilation postoperatively may worsen vascular perfusion of the surgical site and lead to increased risk for anastomotic leakage. NG decompression, which reduces dilation of the esophagus tissue, may serve to reduce the risk for an anastomotic leak [10]. Weijs et al. 2017 meta-analyzed previous controlled trials that showed the early removal of NG tubes (no NG tube or removal within 1-2 postoperative days) did not significantly reduce  incidence of AL compared to prolonged NG tube removal (6 to 10 postoperative days) after esophagectomy [11]. Additional studies have explored the effect of different anastomotic surgical techniques on the incidence AL [10]. A meta-analysis by Beitler et al. 1998 compared the use of stapled compared to hand-sewn anastomoses and demonstrated no significant difference [12]. An up-to-date summary of the literature comparing previously explored interventions is warranted.

There is a need for an up-to-date review comparing the efficacy of all previous interventions designed to prevent AL after esophagectomy. A systematic approach to grading the quality, which can be accomplished using frameworks such as GRADE (Grading of Recommendations, Assessment, Development, and Evaluations), is essential to guide clinical practice recommendations [13]. Properly conducted RCT studies are the gold standard for evaluating the effect of an intervention [15]. Therefore, the present meta-analysis aimed to provide a comprehensive and up-to-date summary of all previous interventional RCTs that sought to reduce esophagogastric AL rates after esophagectomy, as well as provide systematic grading of quality among meta-analyzed interventions.

## Methods

This systematic review and meta-analysis was conducted according to the Preferred Reporting Items for Systematic Reviews and Meta-analyses guidelines (PRISMA checklist) [14]. The protocol is available in the International Prospective Register of Systematic Reviews (CRD42019127181).

### Search strategy

MEDLINE (OVID interface, including In-process and Epub Ahead of Print) and Embase (OVID interface) databases were searched from 1946 to February 2019 (Additional file 1). The literature search results were uploaded and reviewed using Covidence Software (Covidence, Melbourne, Australia).

### Selection criteria

Potentially  eligible studies were screened by title and abstract (stage 1) followed by full-text article screening to assess full eligibility (stage 2). Literature search results and full-text articles that met full eligibility criteria were reviewed independently and in duplicate by two reviewers. Any disagreement was resolved through discussion with a third reviewer. The reasons for exclusion were recorded. RCT studies that evaluated any intervention aiming to reduce the risk of AL after esophagectomy were included with no restriction on language. Only RCT studies that reported AL, our primary outcome, were included Articles including reviews, editorials, preclinical studies, observational studies, and abstracts were excluded.

### Outcome justification and prioritization

Our primary outcome AL was defined as the presence of extraluminal collections of air or contrast, excess bile-stained fluid on drainage, or a combination [15]. Secondary outcomes of interest included anastomotic stricture, mortality, and length of stay in hospital postoperatively.

### Data extraction

Patient and study characteristics, intervention details, and outcomes of interest were extracted from RCT studies that met full eligiblity. Two reviewers performed data extraction independently and in duplicate. Any disagreements were resolved by a third reviewer. The patient characteristics recorded included the total number of patients investigated (intervention and control groups), the mean age of participants (± SD), and the ratio of males-to-females. The study characteristics recorded included the first author name, year of publication, study country of origin, the prevalence of AL (%), and the use of neoadjuvant therapy (e.g. radiation, chemotherapy), the mean (± SD) or median (IQR) length of stay in days, and the mean follow-up in months (± SD). The intervention details recorded included the intervention type (e.g. omentoplasty, stapled vs. hand-sewn anastomosis, early NG tube removal, other), diagnostic modality used for anastomosis (e.g. gastrografin contrast), medical management used (e.g. antibiotics), endoscopic management used (e.g. NG tube use), the surgical management used (e.g. reoperation) and the surgical approach for esophagogastric anastomosis (e.g. cervical or thoracic anastomosis). 

### Summary measures and synthesis of results

Open Meta-Analyst (Open-source, USA) was used to generate heterogeneity measurements and effect estimates for risk ratios (RR) and weighted mean differences (WMD). The pooled (RR) estimates and pooled weighted mean difference (WMD) estimates (evaluating the difference in the length of stay between intervention and control groups)  were determined using dichotomous and continuous DerSimonian and Laird random effects models, respectively [16]. Pooled RR and WMD estimates were stratified according to intervention type. The heterogeneity of pooled effect sizes was assessed using the Cochrane I^2^ statistic and the level was approximated using the following thresholds: 0–40% (low heterogeneity), 30–60% (moderate heterogeneity), 50–90% (substantial heterogeneity) and 75–100% (considerable heterogeneity) [16].  Studies that did not report the mean (± SD) length of stay were excluded from the pooled WMD estimate. The statistical significance was evaluated using a 95% confidence interval (CI). Subgroup analysis of AL was performed according to the type of disease (e.g. esophageal cancer), age (≤ or > 18 years old), type of surgery (cervical vs. thoracic anastomosis), and use of induction therapy or neoadjuvant therapy.

A pooled RR estimate of greater than one represented a higher risk of AL, stricture, or and mortality; a value less than one demonstrated a lower risk. A pooled WMD estimate of less than zero represented a shorter length of stay in the intervention group (vs. control group); a value greater than zero represented a longer length of stay.

### Risk of bias

The Cochrane revised risk of bias tool for randomized trials was used to evaluate the individual risk of bias for studies reviewed [13]. Within each risk of bias domain, a series of questions (‘signaling questions’) were chosen to elicit information about features of the trial that were deemed to be relevant to the risk of bias. Publication bias was included in the assessment. Judgement was classified as 'low', 'high', or as having ‘some concerns' [13]. Meta-bias (risk of bias across studies) was summarized by pooling the individual study risk of bias for each risk of bias domain.

### Grading of recommendations, assessment, development, and evaluations

The quality for each intervention effect was graded by using a systematic and comprehensive approach known as GRADE [13]. GRADE provides a reproducible and transparent framework for grading the quality of evidence or certainty in the evidence. The quality of evidence reflects the extent to which we are confident that an estimate of the effect is correct. High grade of evidence means the true estimate lies close to the estimate of effect; moderate grade means that the true effect is likely to be close to the estimate of the effect; low grade means that the effect estimate may substantially differ from the true estimate of the effect; very low grade means we have little confidence in the effect estimate [13].

## Results

**Fig. 1:**
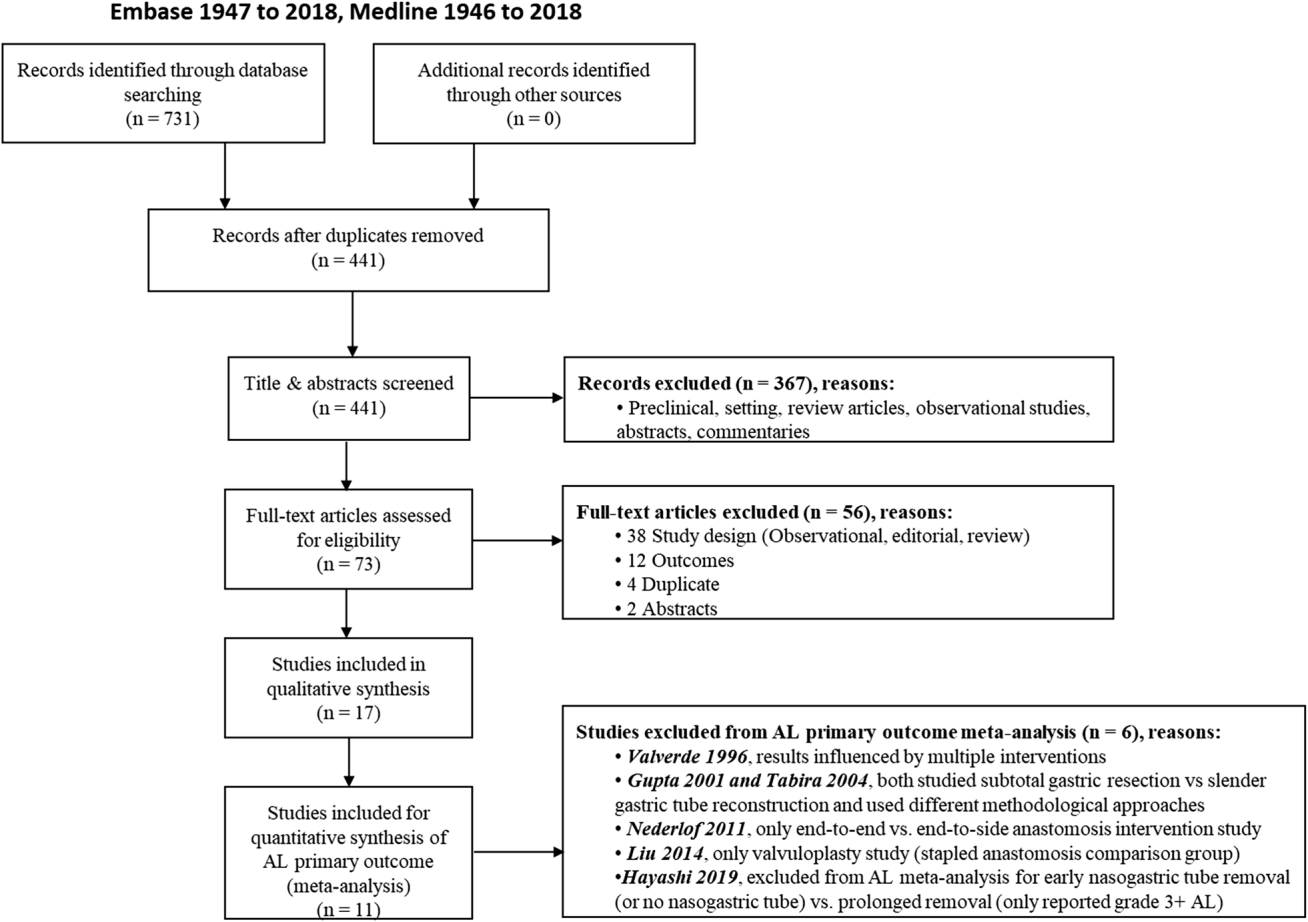
PRISMA flow diagram summarizing screening and selection of eligible studies

The systematic searches returned a total of 731 citations. Following deduplication, 441 citations were identified. Of the 441 citations, 73 full manuscripts were identified as potentially eligible with a total of 17 RCTs meeting our eligibility criteria (n = 3,157 patients). Eleven studies were included in our meta-analysis as shown in our PRISMA flow diagram (Fig. 1).

Studies were published between 1996 and 2019, with sample sizes ranging from 32 to 516 participants. The mean age of participants was similar across studies ranging from 50.8 to 67.5 years old. Follow-up periods were highly variable ranging from 3 months to 3 years. Most studies were performed in China (5 studies, 19%), India (4 studies, 24%), Japan (3 studies, 18%), or other (5 studies [Iran, Hong Kong, Thailand, Netherlands, France], 29%). The proportion of male participants was higher than female participants in all studies except one. The incidence of AL ranged from 1.4 to 17%. The patient characteristics of the included studies are provided in Table 1.

**Table 1:** Characteristics of the included studies and participants studies

First author (year)	Country	Total participants (N)	Total participants in intervention groups (n)	Prevalence AL (%)	Male-to-female ratio evaluated (T, I, C)	Age, years (y) Mean ± SD	Neoadjuvant therapy	Follow-up, months (mo, wk, or y) Mean ± SD
Bhat 2006 [6]	India	194	97	8.8	T: 3:1.8	T: 52.5	Excluded patients with previous neoadjuvant Tx	F/U every 3-mo for 3 y postop every 4- to 6-mo postop
Dai 2011 [5]	China	253	127	3.1	T: 4:01	T: 63.5	Excluded patients with previous neoadjuvant Tx	*22 mo (3–52 mo)
Daryaei 2008 [19]	Iran	40	18	15	NR	T: 58.4 ± 10.3	NP	NP
Gupta 2001 [20]	India	100	48	12	I: 0.8:1 C: 0.7:1	I: 51.3 ± 13.0 C: 50.8 ± 13.2	Rad ± Chemo: 22/100 and Chemo alone: 56/100	3 mo or more
Hayashi 2019 [21]	Japan	71	34	5.6	T: 6:8	T: 63.04	NP	NP
Law 1997 [22]	Hong Kong	122	61	3.3	I: 7.7:1 C: 6.6:1	I: 64 ± 1.2 C: 63 ± 1.0	NP	20 (SD 2.2) mo hand-sewn and 19 (2.2) mo stapled group (p = NS)
Liu 2014 [7]	China	378	188	4.2	T: 3:01	T: 64	Excluded patients with previous neoadjuvant Tx	NP
Liu 2015 [23]	China	432	219	5	I: 3:1 C: 3:1	I: 62 ± 8 C: 61 ± 9	Rad + chemo: 64/478	17.8 (3.2) mo hand-sewn and 18.3 (3.4) mo stapled
Luechakiettisak 2008 [24]	Thailand	104	52	4.8	I: 4.8:1 C: 5.6:1	I: 63.6 C: 62.0	NP	NP
Mistry 2012 [25]	India	253	127	3.1	T: 2.1:1	I: 53.4 C: 56.7	Rad ± Chemo: 2/150 and Chemo alone: 72/150	NP
Nederlof 2011 [26]	Netherlands	128	64	31	I: 2:1 C: 7:1	I: 60 C: 63	Rad + Chemo: 27/64 and Chemo alone: 17/64	3- or 6-wk outpatient visit. 3 mo first y postop. Every 4 mo second y postop
Okuyama 2007 [27]	Japan	32	14	12	I: 13:1 C: 16:2	I: 63.6 C: 64.3	Excluded patients with previous neoadjuvant Tx	5 y
Saluja 2012 [28]	India	174	87	17	I: 2.3:1 C: 1.6:1	I: 51.4 ± 12 C:50.9 ± 14	Rad + Chemo: 107/174	NP
Zhang 2010 [29]	China	516	272	1.4	I: 1.4:1 C: 1.4:1	I: 59 ± 1.2 C: 60 ± 1.3	Excluded patients with previous neoadjuvant Tx	12 mo
Zheng 2013 [4]	China	164	82	8.5	I: 1.6:1 C: 1.4:1	I: 67.5 ± 11.2 C: 65.7 ± 9.4	None of the patients received chemotherapy or radiotherapy pre-op	3 y
Tabira 2004 [30]	Japan	44	22	14	I: 6.3:1 C: 10:1	I: 64 ± 8 C: 60 ± 8	NP	NP
Valverde 1996 [31]	France	152	78	16	I: 9.6:1 C: 10.1:1	I: 59 ± 9 C: 59 ± 10	NP	NP

*C* control, *Chemo* chemotherapy, *I* intervention, *mo* months, *NP* not provided, *pre-op* pre-operatively, *postop* postoperatively, *Rad* radiation, *SD* standard deviation, *Tx* therapy, *wk* weeks, *Y* years

*Median (range)

Seven studies (41%) investigated stapled (vs. hand sewn) anastomosis, three studies (18%) investigated omentoplasty (vs. conventional stapled or hand-sewn anastomosis), three studies (18%) investigated early NG tube removal (postop day 1 or 2 days) or no NG tube (vs. conventional 7 to 10 days to NG tube removal), two studies (12%) that investigated subtotal gastric resection (vs. slender gastric tube) reconstruction, one study (6%) investigated valvuloplasty (vs. stapled anastomosis), and one study (6%) that compared end-to-end (vs. end-to-side) anastomosis. Sixteen studies (94%) used contrast to diagnose AL and six studies (35%) used additional endoscopy and/or chest tube or drain output. Seven studies (41%) administered medical management, three studies (18%) administered surgical management, and two studies (12%) administered endoscopic management for the treatment of AL. The length of stay in hospital postoperatively varied from 10.7 to 29.4 days. The study intervention characteristics are outlined in Table 2.

**Table 2:** Intervention characteristics of the included studies

First author (year)	Intervention and control groups	Surgical approach to intervention	Length of stay, days	Diagnostic modality for anastomosis	Medical management	Endoscopic management	Surgical management
Bhat 2006 [6]	Omentoplasty (I) vs hand-sewn anastomosis alone (C)	Cervical: 102 Thoracic: 92	NP	Water-soluble contrast	Abx, bronchodilators, chest physiotherapy	Re-insertion NG tube	Re-exploration, refashioning anastomosis
Dai 2011 [5]	Omentoplasty (I) vs stapled anastomosis alone (C)	Cervical: 75 Thoracic: 180	I: 20.4 (11.5)** C: 23.1 (15.2)**	Contrast	NP	NP	NP
Daryaei 2008 [19]	NG tube (I) vs Metoclopramide (C)	Cervical: 20 Thoracic: 20	I: 10.9 (3.5** C: 13.9 (8.2)**	Gastrografin contrast	Metoclopramide (C)	NP	NP
Gupta 2001^a^ [20]	Subtotal (I) vs slender anastomosis (C)	Cervical only	I: 10.7 (3.6)** C: 11.9 (5.6)**	Water-soluble contrast	Not reported	NP	NP
Hayashi 2019^a^ [21]	Early NG tube removal or no NG tube (I) vs prolonged NG tube removal (C)	Thoracic only	I: 25.7 (12.76)** C: 29.4 (18.06)**	Contrast agent	All patients received PPI, ICU admission postop	Re-insertion of NG tube	Tracheostomy, mini-tracheostomy
Law 1997 [22]	Stapler (I) vs hand sewn anastomosis (C)	Thoracic only	NP	Gastrografin contrast, endoscopy	NP	NP	NP
Liu 2014 [7]	Valvuloplasty (I) vs stapled anastomosis alone (C)	Cervical: 126 Thoracic: 259	I: 20.4 (11.5)** C: 22.1 (15.2)**	Contrast, endoscopy	NP	NP	NP
Liu 2015 [23]	Stapler (I) vs hand sewn anastomosis (C)	Cervical: 113 Thoracic: 354	I: 20.1 (6.8)** C: 18.9 (7.3)**	Barium swallow, endoscopy	Nutrition, chest tube drain	NP	NP
Luechakiettisak 2008 [24]	Stapler (I) vs hand sewn anastomosis (C)	Thoracic only	NP	Gastrografin contrast	NP	NP	NP
Mistry 2012 [25]	Short-term (I) vs prolonged NG tube (C)	Cervical: 33 Thoracic: 117	I: 12 (9 – 17)* C: 12 (10 – 17)*	Contrast	NP	NP	NP
Nederlof 2011^a^ [26]	End-to-end (I) vs side-to-end (C) anastomosis	Cervical: 88 Thoracic: 40	I: 15 (9 – 125)* C: 22 (8 – 281)*	Contrast, endoscopy, neck wound saliva	NP	NP	Re-operation
Okuyama 2007 [27]	Stapler (I) vs hand sewn anastomosis (C)	Cervical: 18 Thoracic: 14	NP	Water-soluble contrast	Conservative	NP	NP
Saluja 2012 [28]	Stapler (I) vs hand sewn anastomosis (C)	Cervical only	I: 12.8 (8)** C: 11.9 (6)**	Gastrografin contrast	Abx, opening neck wound	NP	NP
Zhang 2010 [29]	Stapler (I) vs hand sewn anastomosis (C)	Thoracic only	NP	Chest tube output, contrast barium, endoscopy	Nutrition, chest tube drain	NP	NP
Zheng 2013 [4]	Omentoplasty (I) vs hand-sewn anastomosis (C)	Thoracic only	I: 21 (5)** C: 23 (6)**	Gastrografin contrast	NP	NP	NP
Tabira 2004 [30]	Subtotal (I) vs slender gastric tube (C)	Thoracic only	NP	NP	Conservative	NP	NP
Valverde 1996^a^ [31]	Stapler (I) vs hand sewn anastomosis (C)	Cervical: 45 Thoracic: 107	NP	Swallow, methylene blue, interstitial fluid in drains	NP	NP	NP

*Abx* antibiotics, *C* control, *I* intervention, *mo* months, *NP* not provided, *POD* postoperative day, *TE* transesophageal, *TH* transhiatal, *TT* transthoracic, *wk* weeks, *Y* years

*Median (IQR)

**Mean (SD)

^a^Excluded from meta-analysis (Valverde 1996, Group results influenced by multiple additional interventions; Nederlof 2011, only study to report intervention type; Hayashi 2019, excluded from AL pooled results because of restriction to reporting grade 3+ AL only; Gupta 2001, only study to report intervention type)

### Primary outcome

#### Anastomotic leak

**Fig. 2:**
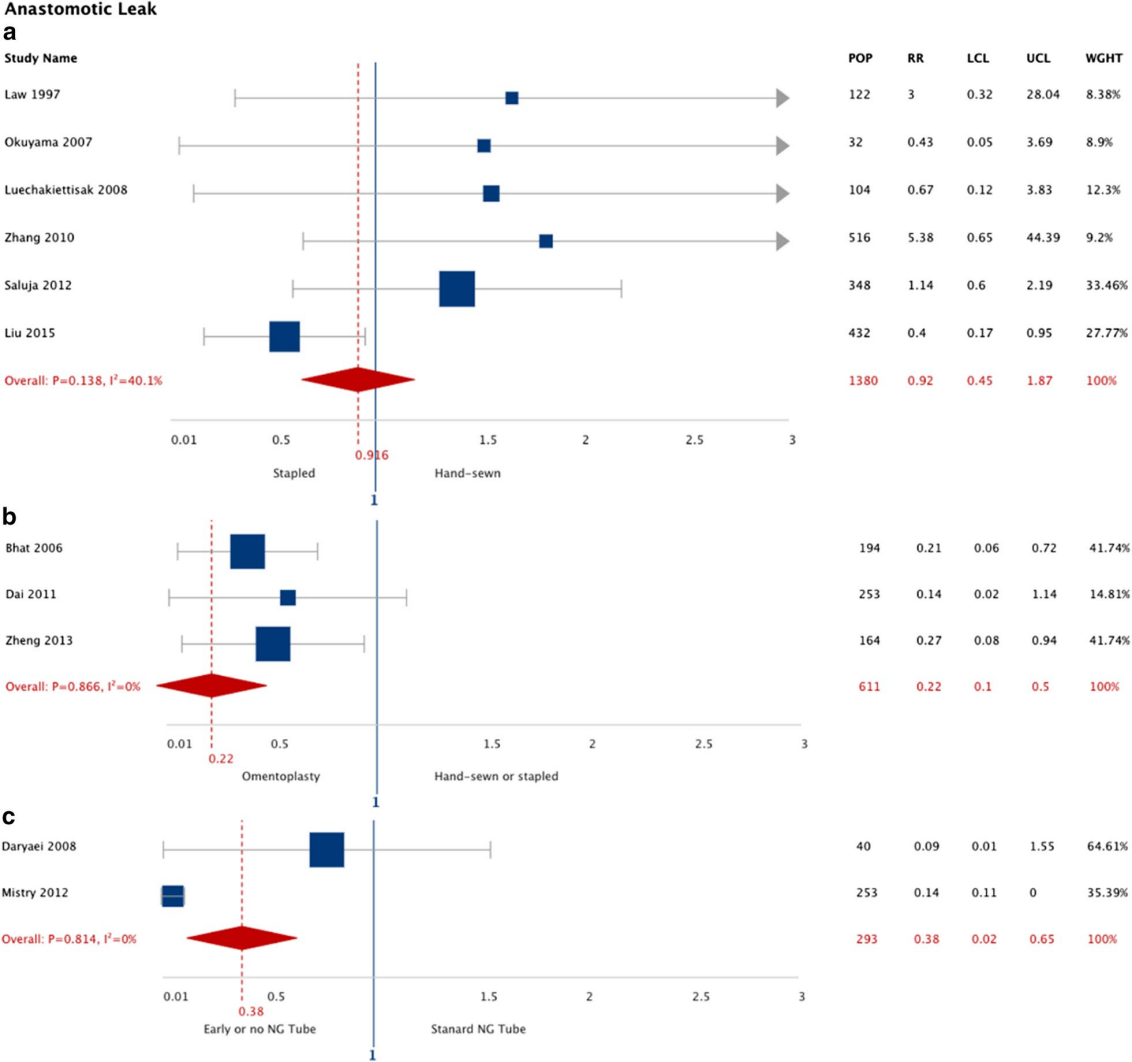
Pooled risk ratio for anastomotic leakage according to intervention type (11 meta-analyzed studies). Stapled anastomosis intervention compared to hand-sewn (**a**). Omentoplasty intervention compared conventional anastomosis (hand-sewn or stapled or hand-sewn anastomosis) (**b**). Early removal nasogastric tube (or no nasogastric tube) intervention compared to prolonged nasogastric tube removal (**c**). *LCL*, lower confidence limit; *UCL*, upper confidence limit; *RR*, risk ratio; *POP*, population size

The pooled results for eleven meta-analyzed studies are summarized in Fig. 2 and the descriptive results for single RCT interventions are summarized in Additional file 1: Table S2. Esophagectomy patients that received stapled esophagogastric anastomosis demonstrated a similar risk of AL (RR: 0.92; 95% CI: 0.45, 1.87; I^2^ 40.1%) compared to hand-sewn that was not significantly different (6 studies, n = 1454 patients). Esophagectomy patients that received omentoplasty had a 78% reduction in risk of AL (RR: 0.22; 95% CI: 0.10, 0.50; I^2^ 0%) compared to hand-sewn or stapled anastomosis alone that was significant (3 studies, n = 611 patients). Esophagectomy patients with early NG tube removal (or no NG tube) demonstrated a 62% reduction in risk of AL (RR: 0.38; 95% CI: 0.02, 0.65; I^2^ 0%) compared to prolonged NG tube removal that was significant (2 studies, n = 293 patients).

The pooled RR estimates for AL were subgrouped according to the site of esophagogastric anastomosis (Table 3). The pooled RR estimate for AL in the cervical esophagogastric anastomosis subgroup (2 studies, RR: 0.23; 95% CI: 0.069, 0.788; I^2^ 0%) was not significantly different compared to the pooled RR for thoracic esophagogastric anastomosis subgroup (2 studies, RR: 0.19; 95% CI: 0.034, 1.032; I^2^ 0%). The pooled RR estimates for AL were also subgrouped according to stapled or hand-sewn esophagogastric anastomosis (Additional file 1: Table S3). The RR estimate for AL in the stapled esophagogastric anastomosis subgroup (1 study, n = 194 patients, RR: 0.214; 95% CI: 0.064, 0.722) was not significantly different compared to the pooled RR estimate in the hand-sewn esophagogastric anastomosis subgroup (2 studies, n = 417 patients, RR: 0.264; 95% CI: 0.089, 0.789). Due to a lack of reporting of AL according to neoadjuvant therapy type (radiation and/or chemotherapy), it was not possible to perform this planned subgroup analysis.

**Table 3:** Risk ratios for anastomotic leak for omentoplasty intervention (subgroup by cervical or thoracic approach)

Group	Study (Author, year)	Risk ratio	95% CI (lower, upper)	I^2^
Cervical*	Bhat 2006^6^	0.22	0.080, 0.88^*^	–
	Dai 2011^5^	0.26	0.030, 2.08	–
	Overall (n = 2 studies)	0.23	0.080, 0.88^*^	0
Thoracic*	Bhat 2006^6^	0.19	0.020, 1.52	–
	Dai 2011^5^	0.18	0.010, 3.68	–
	Overall (n = 2 studies)	0.19	0.030, 1.03	0

*Omentoplasty vs. conventional anastomosis (stapled or hand-sewn anastomosis)

### Secondary outcomes

#### Anastomotic stricture

**Fig. 3:**
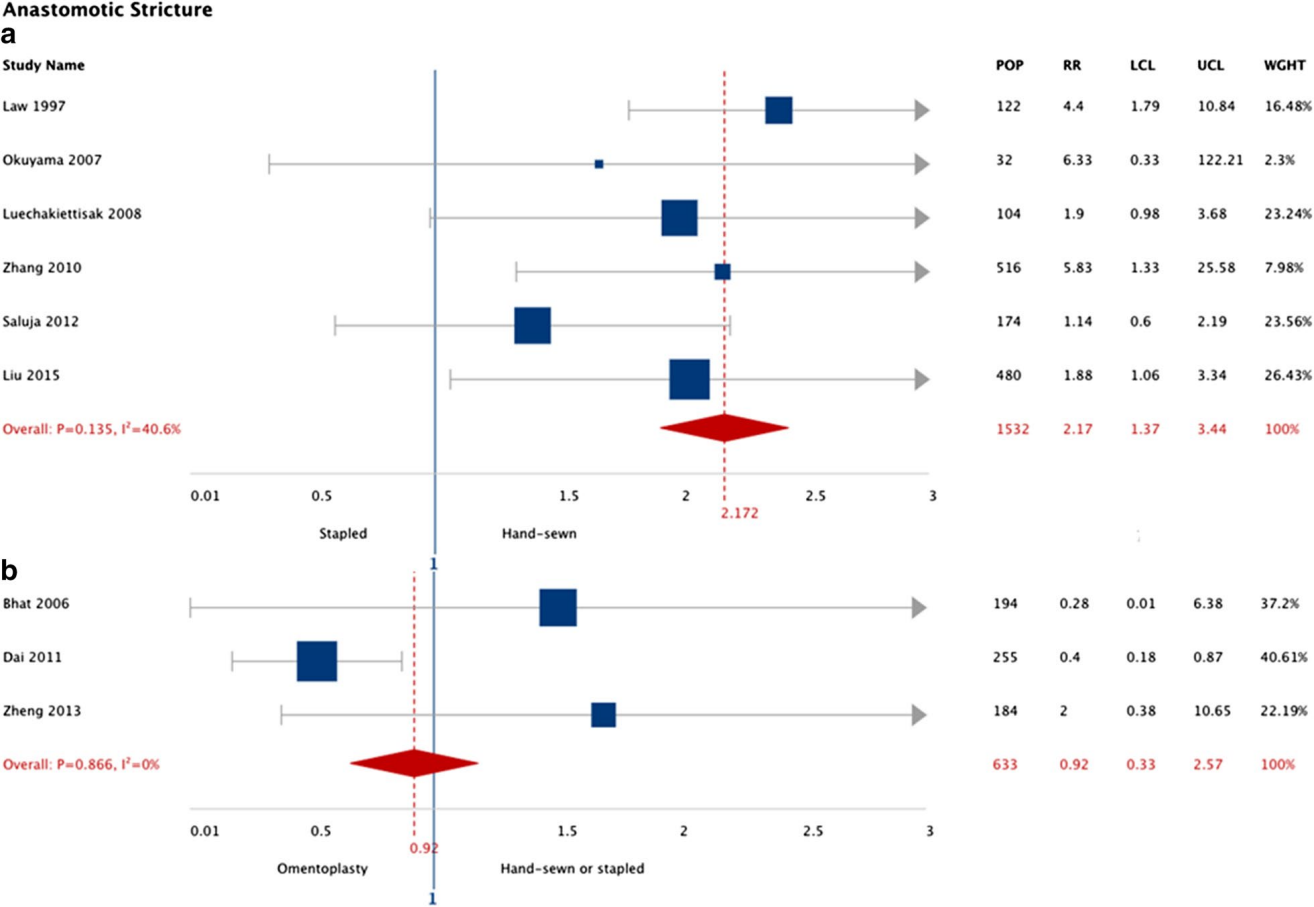
Pooled risk ratio for anastomotic stricture grouped according to intervention type (11 meta-analyzed studies). Stapled anastomosis intervention compared to hand-sewn (**a**). Omentoplasty intervention compared to conventional anastomosis (stapled or hand-sewn anastomosis) (**b**). *LCL*, lower confidence limit; *UCL*, upper confidence limit; *RR*, risk ratio; *POP*, population size

Esophagectomy patients that received stapled esophagogastric anastomosis had a twofold increased risk of stricture (RR: 2.11; 95% CI: 1.36, 3.26; I^2^ 35.0%) compared to hand-sewn (6 studies, n = 1380 patients). Esophagectomy patients that received omentoplasty had an 8% lower risk of stricture (RR: 0.92; 95% CI: 0.33, 2.57; I^2^ 65.1%) that was not significantly different compared to conventional anastomosis (3 studies, n = 613 patients). The pooled results are summarized in Fig. 3.

#### Mortality rate

**Fig. 4:**
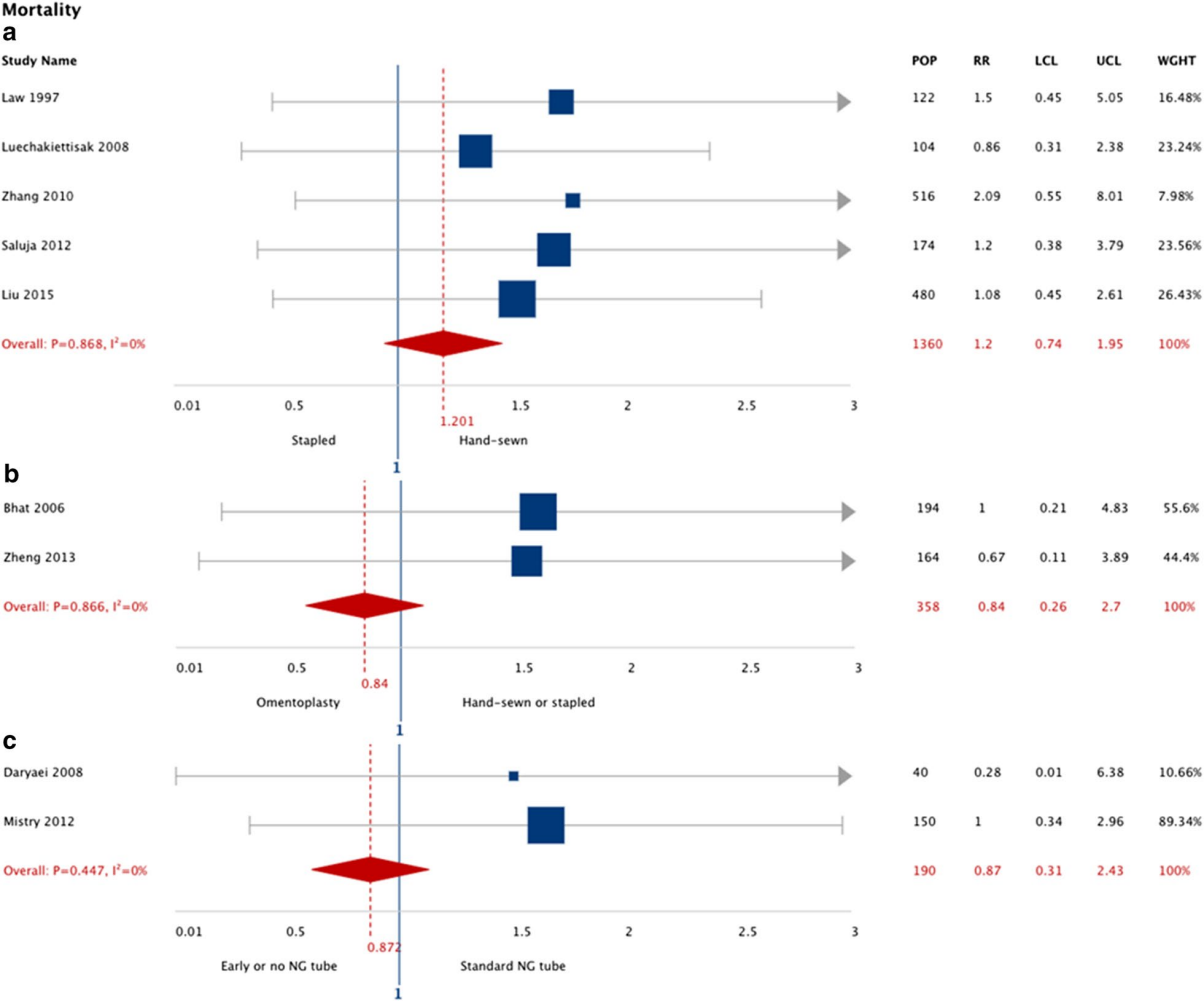
Pooled risk ratio for mortality grouped according to intervention type (11 meta-analyzed studies). Anastomotic stricture grouped by intervention type. Stapled anastomosis intervention compared to hand-sewn (**a**). Omentoplasty intervention compared conventional anastomosis (hand-sewn or stapled or hand-sewn anastomosis) (**b**). Early  nasogastric tube removal (or no nasogastric tube) intervention compared to prolonged nasogastric tube removal (**c**). Overall mortality reported across studies except when marked (*) as 30-day mortality. *LCL*, lower confidence limit; *UCL*, upper confidence limit; *RR*, risk ratio; *POP*, population size

Esophagectomy patients that received stapled esophagogastric anastomosis had no statistically significant difference in risk of mortality (RR: 1.22; 95% CI: 0.75, 1.98; I^2^ 0%) compared to hand-sewn esophagogastric anastomosis (6 studies, n = 1363 patients). Esophagectomy patients that received omentoplasty had a 20% lower risk of mortality (RR: 0.80; 95% CI: 0.32, 2.0; I^2^ 0%) compared to conventional anastomosis (3 studies, n = 736 patients). Esophagectomy patients with early NG tube removal (or no NG tube) demonstrated no statistically significant difference in risk of mortality (RR: 0.90; 95% CI: 0.317, 2.55; I^2^ 0%) compared to prolonged NG tube removal (2 studies, n = 190 patients). The pooled results are summarized in Fig. 4.

#### Length of stay

The pooled weighted mean difference (WMD) for length of stay in hospital postoperatively was determined based on statistical comparison of the mean (± SD) length of stay reported for intervention and control groups among the included studies. Esophagectomy patients that received stapled anastomosis had a 1.1-day longer length of stay in hospital [95% CI: − 0.01, 2.2; I^2^ 0%] compared to hand-sewn anastomosis that was not significantly different (2 studies, n = 606 patients). Esophagectomy patients that received omentoplasty had a 2.1-day shorter length of stay in hospital (WMD: − 2.1; 95% CI: − 3.6, − 0.6; I^2^ 0%) that was statistically significant compared to stapled or hand-sewn anastomosis alone (2 studies, n = 417 patients). Esophagectomy patients with early NG tube removal (or no NG tube) had a 3.2-day shorter length of stay in hospital (WMD: -3.2; 95% CI: − 6.5, 0.2; I^2^ 0%) compared to prolonged NG tube removal that was not significantly different (2 studies, n = 111 patients). Mistry et al*.* 2012 was excluded from the pooled WMD estimate as only median (IQR) was reported for length of stay; early NG tube removal (or no NG tube) and prolonged NG tube removal groups each had a median length of stay of 12 days with similar variability of 9–17 and 10–17 days, respectively (P = 0.18) [17].

#### Risk of bias

**Fig. 5:**
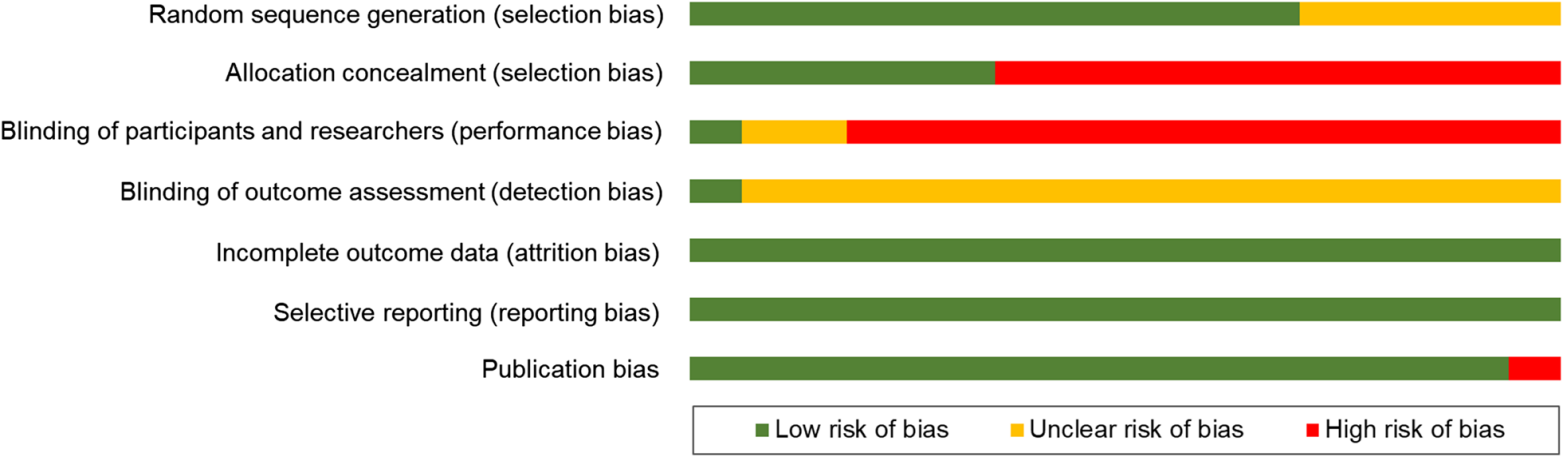
Revised Cochrane risk of bias tool for randomized controlled trial studies included (11 meta-analyzed studies). Green, low risk of bias; yellow, unclear risk of bias; and red, high risk of bias

Seven (64%) meta-analyzed studies did not report whether the allocation of participants was concealed. Nine (82%) meta-analyzed studies lacked any details surrounding blinding of outcome assessment was blinded. Ten (91%) meta-analyzed studies lacked reporting of outcome assessment blinding. The risk of bias across studies is summarized in Fig. 5 (Individual study risk of bias is summarized in Additional file 1: Table S4).

#### Grade

There was a high quality of evidence for AL in the omentoplasty intervention. The unclear risk of bias in omentoplasty studies was due to the lack of allocation concealment in one study decreased the quality of evidence by one level. The large magnitude of effect in the omentoplasty studies increased the quality of evidence by one level. There was a moderate quality of evidence for AL in the early NG tube removal (or no NG tube) intervention. The high risk of bias due to both the lack of randomization and allocation concealment in all studies decreased the quality of evidence by two levels. The large magnitude of effect increased the quality of evidence by one level. There was a very low quality of evidence for AL in the stapled anastomosis intervention. The high risk of bias due to both the lack of randomization and allocation concealment in nearly all studies decreased the quality of evidence by two levels. The imprecision of the measure of effect due to the lack of statistical significance reduced the quality of evidence by one level. The moderate level of heterogeneity in the pooled estimate decreased the quality of evidence by one level. The evidence profile is summarized according to intervention type in Table 4 (GRADE summarized in Additional file 1: Table S5).

**Table 4:** Summary of findings (11 meta-analyzed studies)

Intervention	No. participants (studies)	Quality of evidence	Measure of effect, RR (95% CI)
Omentoplasty vs. conventional anastomosis^a^	611 (3 studies)	+ + + + (high quality) − 1: unclear risk of bias^b^ + 1: large magnitude of effect	RR = 0.22 (78% risk reduction) 95% CI = 0.1, 0.5^*^
Early NG tube removal (or no NG tube) vs. prolonged NG tube removal	374 (2 studies)	+ + + − (moderate quality) − 2: high risk of bias^c^ + 1: large magnitude of effect	RR = 0.38 (62% risk reduction) 95% CI = 0.02, 0.65^*^
Stapled anastomosis vs. hand-sewn anastomosis	1532 (6 studies)	− − − − (very low quality) − 2: high risk of bias^c^ − 1: imprecision in measure of effect^d^ − 1: inconsistency across studies^e^	RR = 0.92 (8% risk reduction) 95% CI = 0.45, 1.87

*RR* risk ratio, *CI* confidence interval, GRADE: working group grades of evidence

High quality (+ +  + +): more research very unlikely to change the estimate of effect

Moderate quality (+ +  + −): means further research is likely to have an important impact on our confidence in the estimate of effect and may alter the estimate

Low quality (+ +− −): means that the effect estimate is limited and may substantially differ from

Very low quality (+−−− or − − − −): grade means that we have little confidence in the effect estimate

*Statistically significant confidence interval

^a^Stapled or hand-sewn anastomosis

^b^One study lacked allocation concealment

^c^Lack of randomization and allocation concealment

^d^Optimal information size not met (Additional file 1: Table S5) and the 95% CI for the effect estimate crosses the null (RR = 1.0)

^e^Moderate heterogeneity (I^2^ = 40.1%)

## Discussion

This is the first systematic review and meta-analysis to provide a graded quality of evidence summary all previous RCT interventions aiming to reduce anastomotic leakage (AL) following esophagectomy. Our review findings suggest there is a high quality of evidence to support omentoplasty as an efficacious intervention to significantly reduce the risk of AL; the perceived benefit that omentoplasty enhances wound healing through increased perfusion to the surgical site offers further justification to this finding [20, 30, 31]. Our findings also showed that omentoplasty lowered the risk of anastomotic stricture, mortality, and length of stay in hospital following esophageal cancer resection, albeit not statistically significantly. The early NG tube removal (or no NG tube) intervention showed a significant reduction in the risk of AL when compared to prolonged NG tube removal, but the moderate quality of evidence for this finding indicates the need for further research. There was a small difference in risk of AL for stapled vs. hand-sewn anastomosis that was not statistically significant and the quality of evidence was very low.

In the present review, subgroup analysis suggested that omentoplasty led to a greater reduction in risk of AL after esophagectomy with a  thoracic anastomosis (transthoracic or Ivor Lewis surgery) compared to cervical anastomosis (transhiatal surgery); but this finding should be interpreted with caution due to the low number of omentoplasty studies reviewed. This finding that  the esophagectomy anastomosis location has an association with the incidence of AL is consistent with previous literature. Studies have shown that patients who undergo transhiatal surgery have a higher incidence of AL compared to transthoracic surgery. However, the possible mechanism(s) explaining this phenomenon remains somewhat controversial [4, 18]. Some studies have attempted to better elucidate the underlying mechanisms. In two previous meta-analyses, the incidence of AL among patients treated with transhiatal esophagectomy  was significantly greater compared to patients treated with transthoracic esophagectomy [30, 32]. Another hypothesis proposes that cervical anastomoses may have higher rates of AL due to perfusion issues of the gastric conduit reaching the neck. The latter is supported by the well-established understanding that perfusion and oxygen delivery to the site of wound healing after surgical resection has a substantial influence on the integrity of the wound healing process [30–32].

There is a need for further research and improved reporting in future studies to allow for elucidation of possible differences in intervention groups. The paucity of studies surrounding the investigation of early NG tube removal (or no NG tube), subtotal gastric reconstruction intervention (vs. a slender tube), and other techniques such as valvuloplasty may reflect the need for further research to better understand the role of these interventions in the prevention of AL. Another limitation in our meta-analysis of omentoplasty studies is that the comparison group included both stapled and hand-sewn anastomosis. However, the risk of AL among omentoplasty studies was not significantly different when subgrouped by stapled vs. hand-sewn anastomosis. Thus, we do not anticipate this limitation to influence our conclusions. Finally, it was not possible to obtain data from the included studies to allow for subgroup analysis according to the use of adjuvant radiation and/or chemotherapy.

Our review identified some gaps in the literature that may be better understood with further research. The lack of reported measures of quality of life or psychometric outcomes was one area where further exploration may be beneficial. The patient-reported outcomes may allow us to better understand key aspects of patient experience to improve the quality of care around the delivery of interventions. Another barrier was the lack of RCT studies performed in North America, which means that our findings may not necessarily be generalizable to North American populations undergoing esophageal cancer treatments. Further research in North American populations may be needed.

## Conclusion

This is the first systematic review and meta-analysis to summarize the efficacy and safety of all previous RCT interventions aiming to reduce anastomotic leakage following esophagectomy. Omentoplasty was found to significantly reduce the risk of anastomotic leak (compared to conventional anastomosis) with a high quality of evidence. Since early (or no) NG tube removal intervention findings provided a moderate quality of evidence, further research is recommended. Future RCTs should aim to strengthen the quality of evidence for this intervention; it demonstrates promising results that are likely to be strengthened by further research. Quality of evidence profiles presented our review may help inform future guideline recommendations surrounding the role of omentoplasty in clinical practice.

## Supplementary Information

**Additional file 1:** Literature search strategy. **Table S2.** Descriptive risk ratio results for RCT’s excluded from meta-analysis**. Table S3.** Risk ratios for anastomotic leak among omentoplasty studies (grouped according to stapled or hand-sewn anastomosis comparison groups). **Table S4.** A revised tool to assess risk of bias in randomized trials (RoB 2). **Table S5.** Grading of Recommendations, Assessment, Development and Evaluations for Anastomotic Leak.

## Data Availability

The dataset generated and analysed in our review are available from the corresponding author on reasonable request.

## Abbreviations

AL: Anastomotic leak
GRADE: Grading of recommendations, assessment, development, and evaluations
IQR: Interquartile range
NG: Nasogastric
PRISMA: Preferred reporting in systematic reviews and meta-analysis
RCT: Randomized controlled trial
RR: Risk ratio
TE: Transesophageal
TH: Transhiatal
TT: Transthoracic
WMD: Weighted mean difference
