# Supplementary material for: Interventions to prevent anastomotic leak after esophageal surgery: a systematic review and meta-analysis
Source: BMC Surg. 2021 Jan 18;21:42. doi: 10.1186/s12893-020-01026-w (PMC7814645; doi:10.1186/s12893-020-01026-w)
Supplement: Supplementary file 1 — Additional file 1: Literature search strategy. Table S2. Descriptive risk ratio results for RCT’s excluded from meta-analysis. Table S3. Risk ratios for anastomotic leak among omentoplasty studies (grouped according to stapled or hand-sewn anastomosis comparison groups). Table S4. A revised tool to assess risk of bias in randomized trials (RoB 2). Table S5. Grading of Recommendations, Assessment, Development and Evaluations for Anastomotic Leak. [file 12893_2020_1026_MOESM1_ESM.docx]

**Supplemental 1.** Literature search strategy

Database: Embase Classic+Embase <1947 to 2018 October 29>, Ovid MEDLINE(R) ALL <1946 to October 29, 2018>, EBM Reviews - Cochrane Central Register of Controlled Trials <September 2018>

Search Strategy:

--------------------------------------------------------------------------------

1 Esophagectomy/ (27375)

2 esophagectom*.tw,kw. (21540)

3 ((esophag* or oesophag*) adj2 resect*).tw. (9650)

4 oesophagectom*.tw,kw. (3900)

5 (oesophag* resect or esophag* resect*).kw. (292)

6 or/1-5 (39269)

7 exp Postoperative Complications/ (1172640)

8 Anastomotic Leak/ or exp Pneumonia/ or exp Arrhythmias, Cardiac/ or Esophageal Stenosis/ (1033298)

9 (Leak* or pneumonia or Arrhythmia* or atri* fibrillat* or stricture* or stenosis).tw,kw. (1277467)

10 7 or 8 or 9 (2913710)

11 6 and 10 (13300)

12 randomized controlled trial.pt. (929319)

13 controlled clinical trial.pt. (183243)

14 random*.tw. (3069515)

15 placebo.ab. (670691)

16 clinical trials as topic.sh. (218326)

17 trial.ti. (683576)

18 or/12-17 (3907711)

19 exp animals/ not humans/ (16931722)

20 18 not 19 (3053793)

21 11 and 20 (974)

22 21 use medall (406)

23 esophagus resection/ (19524)

24 Esophagectom*.tw. (21146)

25 oesophagectom*.tw. (3799)

26 ((esophag* or oesophag*) adj2 resect*).tw. (9650)

27 or/23-26 (36740)

28 exp postoperative complication/ (1172640)

29 anastomosis leakage/ (16096)

30 exp pneumonia/ (376315)

31 exp heart arrhythmia/ (447881)

32 esophagus stenosis/ (8879)

33 (Leak* or pneumonia or Arrhythmia* or atri* fibrillat* or stricture* or stenosis).tw. (1249083)

34 or/28-33 (2783755)

35 27 and 34 (12655)

36 random*.tw. or placebo*.mp. or double-blind*.tw. (3520694)

37 35 and 36 (1195)

38 (exp animal/ or nonhuman/) not exp human/ (11227176)

39 37 not 38 (1174)

40 conference abstract.pt. (3232043)

41 39 and 40 (123)

42 39 not 41 (1051)

43 41 use emczd (116)

44 42 use emczd (498)

45 Esophagectomy/ (27375)

46 esophagectom*.tw,kw. (21540)

47 ((esophag* or oesophag*) adj2 resect*).tw. (9650)

48 oesophagectom*.tw,kw. (3900)

49 (oesophag* resect or esophag* resect*).kw. (292)

50 or/45-49 (39269)

51 exp Postoperative Complications/ (1172640)

52 Anastomotic Leak/ or exp Pneumonia/ or exp Arrhythmias, Cardiac/ or Esophageal Stenosis/ (1033298)

53 (Leak* or pneumonia or Arrhythmia* or atri* fibrillat* or stricture* or stenosis).tw,kw. (1277467)

54 51 or 52 or 53 (2913710)

55 50 and 54 (13300)

56 journal conference abstract.pt. (76813)

57 conference*.pt. (3994578)

58 56 or 57 (4071391)

59 55 and 58 (1969)

60 55 not 59 (11331)

61 59 use cctr (36)

62 60 use cctr (285)

63 22 or 44 or 62 (1189)

64 remove duplicates from 63 (741)

**65 64 use medall (441) MEDLINE**

**Supplemental 2.** Descriptive results for studies excluded from meta-analysis

**Table 3. Descriptive risk ratio results for RCT’s excluded from meta-analysis**

| **Study name** | **Intervention description** | **Risk Ratio (RR, 95% CI)** | | |
| --- | --- | --- | --- | --- |
|  |  | **Anastomotic leakage** | **Anastomotic stricture** | **Mortality** |
| Valverde 1996 | Stapled (I) vs Hand-sewn (C) anastomosis | 0.95 (0.46, 1.98) | 1.0 (0.4, 2.60) | 0.11 (0.01, 2.0) |
| Gupta 2001^ς^ | Subtotal (I) vs Slender gastric tube (C) anastomosis reconstruction | 0.20 (0.047, 0.88)* | 0.29 (0.10, 0.80)* | 1.02 (0.07, 15.90) |
| Tabira 2004^ς^ | Subtotal (I) vs Slender gastric tube (C) anastomosis reconstruction | 0.20 (0.02, 1.6) | NP | 1.0 (0.07, 15.80) |
| Nederlof 2011 | End-to-end (I) vs end-to-side (C) anastomosis | 0.54 (0.31, 0.93)* | 2.50 (1.31, 4.77) | 0.11 (0.010, 2.0) |
| Liu 2014 | Valvuloplasty (I) vs Routine (C) | 0.34 (0.11, 1.03) | 0.53 (0.28, 1.00) | 0.76 (0.172, 3.34) |
| Hayashi 2019 | Early NG decompression (I) vs Routine (C) | 3.62 (0.36, 29.90) | NP | NP |

***Abbreviations:*** NP, not provided

^ς^No meta-analysis because of high variation in study methods for Gupta 2001 and Tabira 2004

**Supplemental 3.**

Risk ratios for anastomotic leak among omentoplasty studies (grouped according to stapled or hand-sewn anastomosis comparison groups)

| **Group** | **Study (Author, year)** | **Risk ratio** | **95% CI (lower, upper)** | **I^2^** |
| --- | --- | --- | --- | --- |
| Omentoplasty vs. Hand-sewn anastomosis | Zheng 2013^4^ | 0.333 | 0.093, 1.192 | - |
|  | Dai 2011^5^ | 0.142 | 0.018, 1.136 | - |
|  | Overall (n = 2 studies) | 0.264 | 0.089, 0.783 | 0 |
| Omentoplasty vs. Stapled anastomosis | Bhat 2006^6^ | 0.214 | 0.064, 0.722 | - |
|  | Overall (n = 1 study) | 0.214 | 0.064, 0.722 | N/A |

**Supplemental 4.** A revised tool to assess risk of bias in randomized trials (RoB 2)

| **Bias** | **Random sequence generation (selection bias)** | **Allocation concealment (selection bias)** | **Blinding of participants and researchers (performance bias)** | **Blinding of outcome assessment (detection bias)** | **Incomplete outcome data (attrition bias)** | | **Selective reporting (reporting bias)** | | **Publication bias** | |  |
| --- | --- | --- | --- | --- | --- | --- | --- | --- | --- | --- | --- |
| Bhat 2006 | Low | HIGH (not reported) | HIGH (reported that the surgeons only knew patient assignment in operating room + did not report whether patients blind) | Some concerns, not reported (but AL is an objective measure so detection bias would likely be minimal) | Low | | Low | | Low | |  |
| Dai 2011 | Low | HIGH (not reported) | Some concerns, only surgeons in operating room not blinded | Some concerns, not reported (but AL is an objective measure so detection bias would likely be minimal) | Low | | Low | | Low | |  |
| Daryaei 2008 | Some concerns (randomization reported, procedure not reported) | HIGH (not reported) | HIGH (reported that the surgeons only knew patient assignment in operating room + did not report whether patients blind) | Some concerns, not reported (but AL is an objective measure so detection bias would likely be minimal) | Low | | Low | | Low | |  |
| Gupta 2001 | Some concerns (randomization reported, procedure not reported) | HIGH (not reported) | HIGH (reported that the surgeons only knew patient assignment in operating room + did not report whether patients blind) | Some concerns, not reported (but AL is an objective measure so detection bias would likely be minimal) | Low | | Low | | Low | |  |
| Hayashi 2019 | Low | HIGH (not reported) | HIGH, did not report blinding | Some concerns, not reported (but AL is an objective measure so detection bias would likely be minimal) | Low | | Low | | HIGH, pharmaceutical company funded | |  |
| Law 1997 | Low | Low | HIGH, did not report blinding | Some concerns, not reported (but AL is an objective measure so detection bias would likely be minimal) | Low | | Low | | Low | |  |
| Liu 2014 | Low | Low | Some concerns, only surgeons in operating room not blinded | Some concerns, not reported (but AL is an objective measure so detection bias would likely be minimal) | Low | | Low | | Low | |  |
| Liu 2015 | Low | Low | HIGH, did not report blinding | Some concerns, not reported (but AL is an objective measure so detection bias would likely be minimal) | | Low | | Low | | Low | |
| Luechakiettisak 2008 | Low | Low | HIGH, did not report blinding | Some concerns, not reported (but AL is an objective measure so detection bias would likely be minimal) | Low | | Low | | Low | |  |
| Mistry 2012 | Low | HIGH (reported no blinding used) | HIGH, did not report blinding | Some concerns, not reported (but AL is an objective measure so detection bias would likely be minimal) | Low | | Low | | Low | |  |
| Nederlof 2011 | Low | Low | HIGH, did not report blinding | Some concerns, not reported (but AL is an objective measure so detection bias would likely be minimal) | Low | | Low | | Low | |  |
| Okuyama 2007 | Some concerns (randomization reported, procedure not reported) | HIGH (reported no blinding used) | HIGH (reported no blinding used) | Some concerns, not reported (but AL is an objective measure so detection bias would likely be minimal) | Low | | Low | | Low | |  |
| Saluja 2012 | Low | HIGH (reported no blinding used) | HIGH (reported no blinding used) | Some concerns, not reported (but AL is an objective measure so detection bias would likely be minimal) | Low | | Low | | Low | |  |
| Zhang 2010 | Some concerns (randomization reported, procedure not reported) | HIGH (reported no blinding used) | HIGH (reported no blinding used) | Some concerns, not reported (but AL is an objective measure so detection bias would likely be minimal) | Low | | Low | | Low | |  |
| Zheng 2013 | Low | Low | Low | Low | Low | | Low | | Low | |  |
| Tabira 2004 | Some concerns (randomization reported, procedure not reported) | HIGH (reported no blinding used) | HIGH (reported no blinding used) | Some concerns, not reported (but AL is an objective measure so detection bias would likely be minimal) | Low | | Low | | Low | |  |
| Valverde 1996 | Low | HIGH (reported no blinding used) | HIGH (reported no blinding used) | Some concerns, not reported (but AL is an objective measure so detection bias would likely be minimal) | Low | | Low | | Low | |  |

**Supplemental 5.** Grading of Recommendations, Assessment, Development and Evaluations for Anastomotic Leak

| **Intervention** | **Limitations in study designs or execution (ROB)** | **Imprecision** | **Inconsistency** | **Indirectness** | **Publication bias** | **Factors that increase quality of effect^1^** | **Quality of Evidence (graded out of 4 levels^*^)** |
| --- | --- | --- | --- | --- | --- | --- | --- |
| Omentoplasty (3 studies) | Unclear risk (-1 level) | Low | Low | Low | Low risk | Large magnitude of effect (+1 level) | High quality |
| Early or no NG tube (3 studies) | High risk (-2 levels) | Low | Low | Low | Low risk | Large magnitude of effect (+1 level) | Moderate quality |
| Stapled anastomosis (6 studies) | High risk (-2 levels) | High (-1 levels)  (optimal information size^2^ not met and 95% CI included the null effect) | High (-1 levels) Due to moderate heterogeneity | Low | Low risk | None | Very low quality |

| GRADE: Working group grades of evidence  **High quality:** More research very unlikely to change the estimate of effect  **Moderate quality:** Means further research is likely to have an important impact on our confidence in the estimate of effect and may alter the estimate  **Low quality:** means that the effect estimate is limited and may substantially differ from  **Very low quality:** grade means that we have little confidence in the effect estimate |
| --- |

^1^Large magnitude of effect (increase 1 or 2 levels); All plausible confounding would reduce the demonstrated effect or increase the effect if no effect was observed (increase 1 level); Dose-response gradient (increase 1 level)

^2^Optimal information size calculated using the pooled incidence rates for patients who received the stapled anastomosis intervention and patients who received the hand-sewn anastomosis, 5% and 5.8% respectively. An α 0.05, β 0.2, and a ratio of 1:1 for the comparison groups were used. The optimal information size was determined to be 12529 per group (or 25058 total), which was not met.
